# Population genetic structure of *Schistosoma haematobium* and *Schistosoma haematobium* × *Schistosoma bovis* hybrids among school-aged children in Côte d’Ivoire

**DOI:** 10.1051/parasite/2022023

**Published:** 2022-05-03

**Authors:** Etienne K. Angora, Alexane Vangraefschepe, Jean-François Allienne, Hervé Menan, Jean T. Coulibaly, Aboulaye Meïté, Giovanna Raso, Mirko S. Winkler, William Yavo, André O. Touré, Eliézer K. N’Goran, Jakob Zinsstag, Jürg Utzinger, Oliver Balmer, Jérôme Boissier

**Affiliations:** 1 Swiss Tropical and Public Health Institute P.O. Box CH-4002 Basel Switzerland; 2 University of Basel Kreuzstrasse 2 CH-4123 Allschwil Switzerland; 3 Unité de Formation et de Recherche Sciences Pharmaceutiques et Biologiques, Université Félix Houphouët-Boigny BPV 34 Abidjan Côte d’Ivoire; 4 IHPE, Univ. Montpellier, CNRS, Ifremer, Univ. Perpignan Via Domitia 66860 Perpignan France; 5 Unité de Formation et de Recherche Biosciences, Université Félix Houphouët-Boigny 22 BP 770 Abidjan 22 Côte d’Ivoire; 6 Centre Suisse de Recherches Scientifiques en Côte d’Ivoire 01 BP 1303 Abidjan 01 Côte d’Ivoire; 7 Programme National de Lutte contre les Maladies Tropicales Négligées à Chimiothérapie Préventive 06 BP 6394 Abidjan 06 Côte d’Ivoire; 8 Institut Pasteur de Côte d’Ivoire BPV 490 Abidjan Côte d’Ivoire

**Keywords:** Côte d’Ivoire, Microsatellites, Population genetics, *Schistosoma haematobium*, Schistosomiasis

## Abstract

While population genetics of *Schistosoma haematobium* have been investigated in West Africa, only scant data are available from Côte d’Ivoire. The purpose of this study was to analyze both genetic variability and genetic structure among *S. haematobium* populations and to quantify the frequency of *S. haematobium* × *S. bovis* hybrids in school-aged children in different parts of Côte d’Ivoire. Urine samples were subjected to a filtration method and examined microscopically for *Schistosoma* eggs in four sites in the western and southern parts of Côte d’Ivoire. A total of 2692 miracidia were collected individually and stored on Whatman^®^ FTA cards. Of these, 2561 miracidia were successfully genotyped for species and hybrid identification using rapid diagnostic multiplex mitochondrial *cox1* PCR and PCR Restriction Fragment Length Polymorphism (PCR-RFLP) analysis of the nuclear *ITS2* region. From 2164 miracidia, 1966 (90.9%) were successfully genotyped using at least 10 nuclear microsatellite loci to investigate genetic diversity and population structure. Significant differences were found between sites in all genetic diversity indices and genotypic differentiation was observed between the site in the West and the three sites in the East. Analysis at the infrapopulation level revealed clustering of parasite genotypes within individual children, particularly in Duekoué (West) and Sikensi (East). Of the six possible *cox1*-*ITS2* genetic profiles obtained from miracidia, *S. bovis cox1* × *S. haematobium ITS2* (42.0%) was the most commonly observed in the populations. We identified only 15 miracidia (0.7%) with an *S. bovis cox1* × *S. bovis ITS2* genotype. Our study provides new insights into the population genetics of *S. haematobium* and *S. haematobium* × *S. bovis* hybrids in humans in Côte d’Ivoire and we advocate for researching hybrid schistosomes in animals such as rodents and cattle in Côte d’Ivoire.

## Abstract (fr)

Alors que la génétique des populations de *Schistosoma haematobium* a été étudiée en Afrique de l’Ouest, seules quelques données sont disponibles pour la Côte d’Ivoire. Le but de cette étude était d’analyser à la fois la variabilité génétique et la structure génétique des populations de *S. haematobium* et de quantifier la fréquence des hybrides *S. haematobium* × *S. bovis* chez les enfants d’âge scolaire dans différentes régions de la Côte d’Ivoire. Des échantillons d’urine ont été soumis à une méthode de filtration et examinés au microscope pour les œufs de *Schistosoma* dans quatre sites de l’ouest et du sud de la Côte d’Ivoire. Au total, 2 692 miracidia ont été collectés individuellement et stockés sur des cartes Whatman^®^ FTA. Parmi ceux-ci, 2 561 miracidia ont été génotypés avec succès pour l’identification des espèces et des hybrides à l’aide de la PCR multiplex de diagnostic rapide du *cox1* mitochondrial et d’une analyse du polymorphisme de longueur des fragments de restriction de PCR (PCR-RFLP) de la région *ITS2* de l’ADN nucléaire. Sur 2 164 miracidia, 1 966 (90,9 %) ont été génotypés avec succès en utilisant au moins 10 loci microsatellites nucléaires pour étudier la diversité génétique et la structure de la population. Des différences significatives ont été trouvées entre les sites dans tous les indices de diversité génétique et une différenciation génotypique a été observée entre le site de l’Ouest et les trois sites de l’Est. L’analyse au niveau de l’infrapopulation a révélé un regroupement des génotypes de parasites au sein de chaque enfant, en particulier à Duekoué (Ouest) et Sikensi (Est). Parmi les six profils génétiques *cox1-ITS2* possibles obtenus à partir de miracidia, *S. bovis cox1* × *S. haematobium ITS2* (42,0 %) était le plus fréquemment observé dans les populations. Nous avons identifié seulement 15 miracidia (0,7 %) avec un génotype *S. bovis cox1* × *S. bovis ITS2*. Notre étude apporte de nouvelles connaissances sur la génétique des populations de *S. haematobium* et des hybrides *S. haematobium* × *S. bovis* chez l’homme en Côte d’Ivoire et nous plaidons pour la recherche de schistosomes hybrides chez les animaux (rongeurs et bovins) en Côte d’Ivoire.

## Introduction

Schistosomiasis is a chronic neglected tropical disease caused by trematodes belonging to the genus *Schistosoma* [14, 40]. The disease affects both humans and animals and is of considerable public health and veterinary concern, particularly in tropical and subtropical zones. According to the World Health Organization (WHO), schistosomiasis is transmitted in over 78 countries and territories throughout a wide belt of the tropics and subtropics [68]. More than 250 million people are infected, mostly in Africa [31], and the global burden of schistosomiasis was estimated at 1.4 million disability-adjusted life years (DALYs) in 2017 [26]. Six species of schistosomes can infect humans: *S. guineensis*, *S. haematobium*, *S. intercalatum* and *S. mansoni* in Africa and the Arabian Peninsula [60], and *S. mekongi* and *S. japonicum* in Asia; *S. mansoni* mainly occurs in Africa, but is also found in Brazil and some Caribbean islands [51]. *Schistosoma haematobium* causes the urogenital form of the disease. Classified as a group I carcinogen, urogenital schistosomiasis can lead to squamous-cell carcinoma of the bladder [35]. In most countries, while human schistosomiasis is well documented, little is known about the prevalence and transmission dynamics of animal schistosomiasis.

In Côte d’Ivoire, both *S. haematobium* and *S. mansoni* are endemic, causing urogenital and intestinal schistosomiasis, respectively [13]. The former is predominant in the central and southern parts [15, 56], while the latter is mainly found in the western parts of Côte d’Ivoire [7, 47, 59]. We recently reported high prevalence of *S. haematobium* in school-aged children in South Côte d’Ivoire [5]. Limited data are available on animal-infecting schistosomes, such as *S. bovis*, a parasite of livestock and rodents. A previous study on post-mortem examinations of cattle in North Côte d’Ivoire reported a prevalence of 35% [1]. These findings were confirmed in a recent cross-sectional survey conducted in different parts along a transect from North to South Côte d’Ivoire, with the highest prevalence of *S. bovis* found in cattle in the northern parts of the country [39].

Habitat change and migration of hosts, which is also linked to climate change, can influence the epidemiology and distribution of schistosomiasis and enhance the occurrence of interspecies hybridization between human and animal schistosomes [38]. *Schistosoma haematobium* × *S. bovis* hybrids are well documented in West Africa, particularly in Benin, Mali, Niger and Senegal [34, 42], with occurrences also reported in Malawi [66] and Corsica, France [9, 10]. More generally, genomic studies have highlighted the fact that several natural strains that were initially identified as “pure” *S. haematobium* are in fact introgressed to some extent with *S. bovis* [44, 48]. Recently, hybrids from miracidia (although not thought to be viable) have been identified in Côte d’Ivoire between *S. haematobium* and *S. mansoni* excreted by humans [17, 41], and between *S. haematobium* and *S. bovis* being transmitted by freshwater snails of the genus *Bulinus* [58] and also from humans [6].

Population genetic structure and genetic diversity varies by *Schistosoma* species [49]. Among the African species, *S. mansoni* is the most widely studied. Several investigations have shown high genetic diversity and strong genetic structure in several countries, including Ethiopia, Kenya, Senegal and Uganda [2–4, 57, 61, 63]. In contrast, studies on *S. haematobium* have shown both less genetic diversity and less population structure compared to *S. mansoni* [30, 46]. This difference in genetic structuration between *S. mansoni* and *S. haematobium* is also apparent at the continental scale [30, 62, 63]. Population genetics data for *S. bovis* are even more limited. One study has investigated the population genetic patterns of *S. bovis* in Cameroon revealing an intermediate pattern with high genetic diversity (i.e. like in *S. mansoni*), but no genetic structuration (i.e. like in *S. haematobium*) at the country scale [18]. At the continental scale, a comparative genomic study revealed a significantly higher genomic diversity between *S. bovis* lineages than between *S. haematobium* lineages [48]. More recently, a study analyzed the genetic patterns of parasites collected from humans and animals in North Senegal and molecularly characterized both microsatellite markers and hybrid genotyping [11]. The authors demonstrated (i) clear genetic separation between parasites recovered from animals compared to those from humans; (ii) no genetic structuration between hybrids and pure parasites from human hosts; and (iii) significant genetic differentiation between different villages of the Senegal River basin. These authors concluded that, in Senegal, animals do not represent a real reservoir for human schistosomiasis and also, there is no cross-over of transmission between humans and animals. However, this conclusion contradicts recent observations from Benin, where *S. haematobium* × *S. bovis* hybrids were found in cows [54] and rodents [55].

The purpose of this study was to investigate the population genetic structure and genetic diversity of *S. haematobium* and *S. haematobium* × *S. bovis* hybrids that were obtained from human urine samples, in four sampling sites of Côte d’Ivoire. We molecularly characterized individual miracidia, using *cox1* and *ITS2* markers to identify *S. haematobium* and *S. haematobium* × *S. bovis* hybrids within the different populations, alongside microsatellite markers [65] to analyze population genetic diversity and structure.

## Materials and methods

### Ethics statement

Ethical clearance was obtained from the Ministère de la Santé et de l’Hygiène Publique en Côte d’Ivoire (reference no. 003–18/MSHP/CNER-kp). School authorities, teachers, parents/guardians and children were informed about the objectives, procedures and potential risks and benefits of the study. Written informed consent was obtained from children’s parents or legal guardians, while children provided oral assent. After sampling, a praziquantel treatment (40 mg/kg) was offered to children found with a *Schistosoma* infection.

### Study sites and collection of miracidia

**Figure 1:**
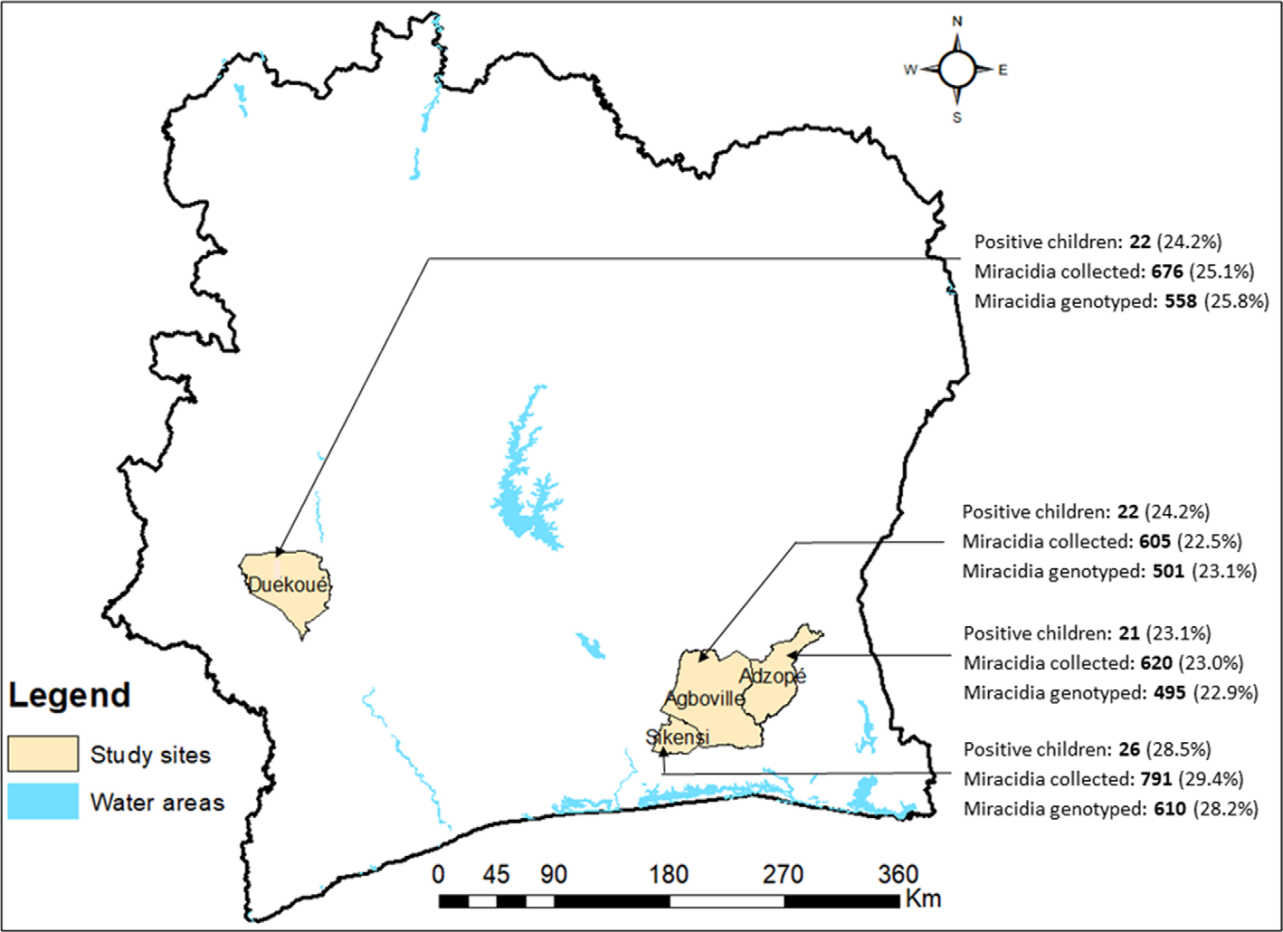
Sites in the southern and western parts of Côte d’Ivoire and number (percentage of total sample size) of *Schistosoma*-infected children, miracidia collected, and miracidia genotyped.

This study was carried out in four sampling sites in Côte d’Ivoire: (i) Agboville (geographical coordinates 5° 55′ 41″ N latitude, 4° 13′ 01″ W longitude) and (ii) Adzopé (6° 06′ 25″ N, 3° 51′ 36″ W) in the south-eastern part; (iii) Sikensi (5°40′34″ N, 4°34′ 33″ W) in the south-central part; and (iv) Duekoué (6° 44′ 00″ N, 7° 21′ 00″ W) in the western part of Côte d’Ivoire (Fig. 1). The study was integrated into a cross-sectional survey of the prevalence of schistosomiasis among school-aged children from January to April 2018 [5]. *Schistosoma* miracidia from children aged 5–14 years were collected, after egg hatching, and individually stored on Whatman^®^ FTA cards (GE Healthcare Life Sciences; Amersham, UK) as previously described [10]. FTA cards were dried for 1 h at room temperature before being stored in a sealed plastic bag and then transferred to the “Interactions Hôtes-Pathogènes-Environnements” (IHPE) laboratory in Perpignan, France, for molecular analysis.

### DNA extraction for miracidia genotyping

Genomic DNA from individual miracidia was extracted using Chelex^®^ beads (Bio-Rad; Hercules, CA, USA) from 2.0 mm discs containing sample that had been punched out of the FTA card using a Harris-Micro-Punch (VWR; London, UK [37].

### *Cox1* and *ITS* analysis for species and hybrid identification

**Supplementary Fig. S1:**
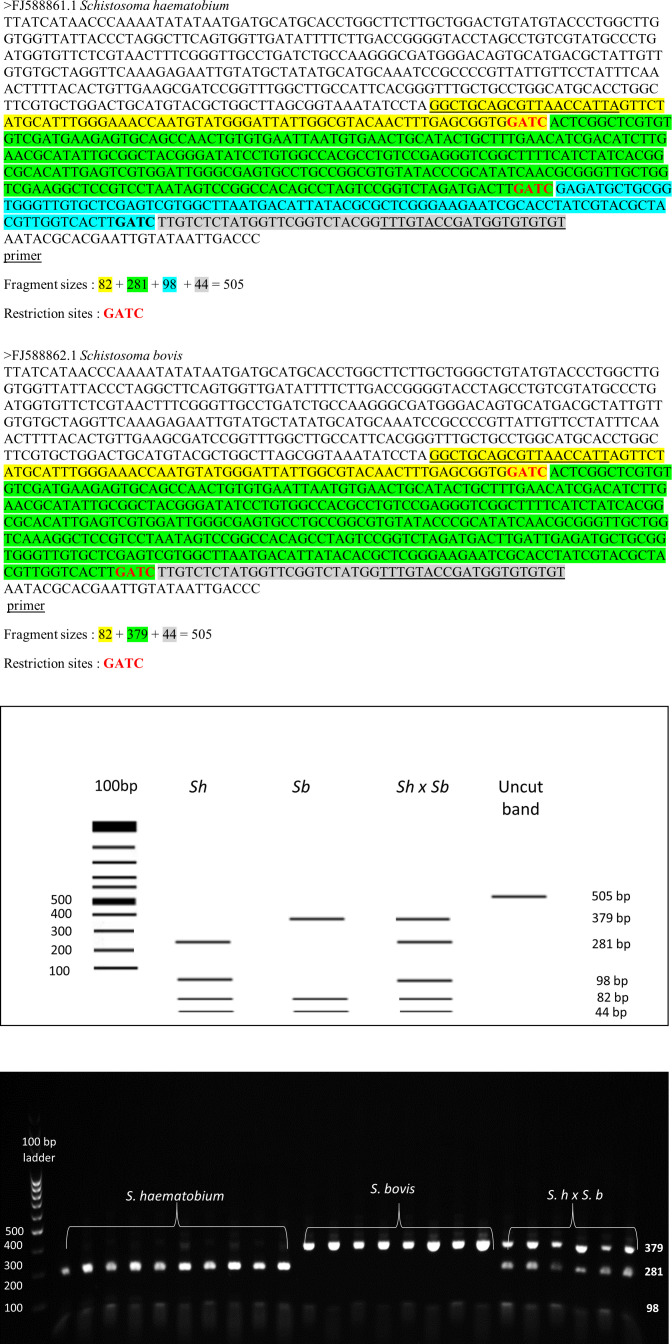
*S1a*: Diagram of the 505 bp fragment and the Mbo1 cut sites for both species, *S1b*: Banding patterns of the restriction fragment length polymorphism (RFLP) analysis by MboI of the nuclear *ITS2* region of *Schistosoma*. *Schistosoma haematobium* exhibits four bands at 281, 98, 82 and 44 bp. *Schistosoma bovis* exhibits three bands at 379, 82 and 44 bp. Hybrids between *S. haematobium* × *S. bovis* exhibit bands at 379, 281, 98, 82 and 44 bp.

A *cox1* multiplex PCR was performed to identify the species-specific mitotype of each sample, as described in our previous work [6]. For the analysis of the nuclear internal transcribed spacer 2 (*ITS2*) region, we used a restriction fragment length polymorphism (RFLP) approach ([19] and Supplementary Fig. S1), following amplification using the forward primer Sc_ITS_F: 5′–GGC TGC AGC GTT AAC CAT TA–3′ and reverse primer Sc_ITS_R: 5′–ACA CAC ACC ATC GGT AC AAA–3′, which targets 505 bp of the *ITS2* [19]. We performed PCRs in a total reaction volume of 25 μL, comprising 2 μL of gDNA, 5 μL of Green GoTaq flexi buffer 5×, 1.5 μL of 25 mM MgCl_2_, 0.5 μL of 10 mM dNTP mix, 1 μL of each 10 μM primer and 1 U of GoTaq Hot Start Polymerase (Promega; Madison, WI, USA). The reaction conditions included an activation step of 95 °C for 3 min, followed by 45 cycles of 95 °C for 40 s, 58 °C for 40 s and 72 °C for 40 s, and a final extension at 72 °C for 6 min. In the subsequent step, 5 μL of PCR products were then digested at 37 °C using 0.5 μL of restriction enzyme MboI (Thermo Fisher Scientific; Waltham, MA, USA), 2.5 μL of CutSmart buffer, and 17 μL of molecular water for 15 min, followed by inactivation at 60 °C for 20 min for a total reaction volume of 25 μL. The cut sites of the MboI enzyme for species specific SNPs were based on the following sequences positions: ↓GATC and CTAG↑. The PCR products were not cleaned or visualized before enzyme digestion. We used only samples that were positive with the *cox1* multiplex PCR method. This enzymatic digestion cuts the 505 bp *ITS2* fragment into four bands for *S. haematobium* (281 bp, 82, bp, 98 bp and 44 bp) and three bands for *S. bovis* (379 bp, 82 bp and 44 bp). All digested *ITS2* PCR products were visualized using 2% agarose electrophoresis gels stained with GelRed^TM^ (Biotium Inc.; Darmstadt, Germany). The nuclear *ITS2* and mitochondrial *cox1* identities for each individual miracidia were combined to produce a mito-nuclear genetic profile of *S. haematobium* (*Shcox1* + *ShITS2*: *Sh* × *ShSh*), *S. bovis* (*Shcox1* + *ShITS2*: *Sb* × *SbSb*) or *S. haematobium-bovis* hybrid genotypes, the latter of which is recognized by mito-nuclear discordance (*Shcox1* + *SbITS2*: *Sh* × *SbSb*), (*Shcox1* + *Sb/ShITS2*: *Sb* × *SbSh*), (*Sbcox1* + *ShITS2*: *Sb* × *ShSh*) or (*Sbcox1* + *Sh/SbITS2*: *Sb* × *ShSb*)).

### Microsatellite analysis

Individual miracidia were further genotyped using a set of 18 microsatellite markers divided into two panels developed by Webster and colleagues [65]. The forward primers were fluorescently labelled with 6-FAM, VIC, NED and PET dyes (Applied Biosystems; Foster City, CA, USA) to enable identification within the multiplex PCR, as previously described [65]. Microsatellite PCRs were performed, using a Microsatellite PCR Kit (Qiagen; Hilden, Germany), in a final volume of 10 μL, including 4 μL of the DNA template, 5 μL of the 2× microsatellite PCR Buffer Kit (Qiagen; Hilden, Germany) and 1 μL of 10× microsatellite primer mix in two PCR panels of nine per multiplex. Thermal cycling was performed with an initial hot-start activation of 15 min at 95 °C, followed by 45 cycles of 94 °C for 30 s, 56 °C for 90 s and 72 °C for 60 s, with a final extension at 60 °C for 30 min. The PCR products were sent to Genoscreen (Lille, France) for genotyping. All microsatellite loci were visually peak called, using GS500Liz size standard (Applied Biosystems), and GeneMarker software. Only 16 loci were subsequently used for analysis as allelic dropout of two loci (C131 and Sh8) occurred in at least 20% of the samples (Supplementary Tab. S1). Errors due to large allele dropout or stutter bands and evidence of the presence of null alleles at each locus were checked using Micro-checker, version 2.2.3 [43].

### Genetic diversity

The number and percentages of the different *cox1*/*ITS2* profiles obtained from each miracidium were calculated for each site and any differences using a χ^2^ test was observed. We also calculated the number of *S. bovis* and *S. haematobium* haplotypes for *cox1* or alleles for *ITS2* among sites. The mitochondrial *cox1* haplotype is unique as it is inherited from the maternal line, while the *ITS2* was scored as one for heterozygous (Sb/Sh) and two for homozygous (SbSb or ShSh). Differences in the relative frequencies of *S. bovis* vs. *S. haematobium* haplotypes or alleles between sites were tested using a binomial test [32].

For the microsatellite data, tests for deviation from Hardy–Weinberg equilibrium per locus and site were carried out using Genepop, version 4.0 [53]. The genotypic disequilibrium test for pairs of loci overall and the adjusted *p*-value for 5% nominal level was performed using FSTAT, version 2.9.4 [28, 67]. Genetic diversity was compared between sites and between *cox1*/*ITS2* genetic profiles. Expected heterozygosity (*He*), number of alleles (*A*), allelic richness (*Ar*), and the inbreeding coefficient (*F*_IS_) of each microsatellite locus were computed per sampling site, using FSTAT version 2.9.4 [28]. *He* and *Ar* were compared between the populations using the pairwise Friedman rank test, followed by the Nemeyi multiple comparison test implemented in the R studio PCMP plus package [36].

### Genotypic differentiation and population structure

Genotypic differentiation between sampling sites was assessed using pairwise *F*_ST_ values [63], calculated in FSTAT, version 2.9.4 [28, 67] with a threshold of significance adjusted for multiple tests using Bonferroni’s standard correction [50]. Pairwise *F*_ST_ values were also calculated between samples that exhibited the different *cox1/ITS2* profiles. Principal component analysis (PCA) was performed to compare either the four sites or the six *cox1/ITS2* profiles, using Genetix, version 4.05 [8].

The uppermost level of genetic structure for all individuals was determined by a Bayesian clustering approach using Markov Chain Monte Carlo (MCMC) permutation analysis, implemented in Structure, version 2.3 [45], using data of children from whom we collected at least 15 miracidia. The length of the burn-in period was 250,000 with the number of MCMC replicates after burn-in at 1,000,000 and *K* from 1 to 8 using an admixture model. The log likelihood for each *K* was averaged over three runs with the CorrSieve package in R (Vienna, Austria) and the delta *K*-values were then computed to determine the most likely number among the *K* values tested [21]. For the most likely number of genetic clusters, an additional 10 runs were computed with the same initial parameters as those described. The probability of each miracidium belonging to each cluster was averaged over the 10 runs and graphically represented using Clumpp, version 1.1.2 [23] and Distruct, version 1.1 [52].

Analysis of molecular variance (AMOVA) was performed to evaluate the partitioning of the overall genetic variance to the hierarchical levels at infrapopulation “within miracidia”, “among miracidia within individual children”, “between miracidia from individual children within each site” and “between sites” using Arlequin, version 3.5 [22]. The level “within miracidia” reflects the differences between microsatellite loci and has no population genetic meaning. Hence, we report the residual variance of the remaining three levels and normalize their relative contributions to 100%.

Population genetic structure between parasite infrapopulation was assessed independently for each sampling site using Structure, version 2.3 with the same procedure as described above. If no best *K* was found, *K* was set as the number of children at each sampling site (16 for Adzopé, 18 for Agboville, 18 for Duekoué and 22 for Sikensi). We first calculated the mean probabilities assignations of the miracidia from a given child to a given cluster. Then repartitions of the miracidia among the different clusters were tested in each participant using χ^2^ under the null hypothesis of random repartition (1/*K*).

## Results

### *Schistosoma haematobium* and hybrid genotype identification

Of the 2692 miracidia collected from the urine samples from 91 *Schistosoma* egg-positive children at four sampling sites, 2561 were genetically analysed. *cox1* and *ITS2* profiles were obtained from 2164 individual miracidia (495 for Adzopé, 501 for Agboville, 610 for Sikensi and 558 for Duekoué) (Fig. 1). Of the 91 positive children, 89 (97.8%) were producing miracidia with a hybrid genotype. Among all possible hybrid profiles, the *S. bovis cox1* × *S. haematobium ITS2* (Sb × ShSh) genotype was the most frequently found in Adzopé (64.6%), Duekoué (56.3%) and Sikensi (30.2%), while the *S. haematobium cox1* × *S. haematobium/S. bovis ITS2* (Sh × ShSb) was the most common hybrid genotype in Agboville (27.7%). We also identified 15 miracidia (0.7%), from seven children, with the *S. bovis cox1* × *S. bovis ITS2* (Sb × SbSb) genetic profile. Overall, 791 miracidia (36.6%) presented an *S. haematobium cox1* × *S. haematobium ITS2* (Sh × ShSh) genetic profile. The χ^2^ test showed a significant difference in the distribution of miracidia with the different *cox1*-*ITS2* profiles between sites (Table 1). The frequency of the *S. bovis cox1* (50.7%) haplotype was similar to that of *S. haematobium* (49.3%; *p* = 0.27). The frequency of the *S. bovis ITS2* allele (12.1%) was statistically lower than the *S. bovis* × *S. haematobium* allele (87.9%; *p* < 0.001). Analysis of the multiplicity of infection showed that 52 children (71.1%) were infected by worm-laying miracidia with at least three different *cox1*-*ITS2* genetic profiles. One child (Id: AG122) from Agboville was infected by adult worms laying miracidia with all six possible *cox1*-*ITS2* genetic profiles (Supplementary Tab. S2).

**Table 1:** Total number (*n*) and percentage (%) of the six possible *cox1-ITS2 genetic* profiles identified using the haploid mitochondrial *cox1* gene (first two letters) and the diploid nuclear *ITS2* region (last four letters), i.e. “pure” *Schistosoma haematobium* (*S. haematobium cox1* × *S. haematobium ITS2*: Sh × ShSh), *S. bovis* genetic signature (*S. bovis cox1* × *S. bovis ITS2*: Sb × SbSb) and four types of hybrid (*S. bovis cox1* × *S. haematobium ITS2*_*S. bovis ITS2*: Sb × ShSb; *S. bovis cox1* × *S. haematobium ITS2*: Sb × ShSh; *S. haematobium cox1* × *S. bovis ITS2*: Sh × SbSb; *S. haematobium cox1* × *S. bovis ITS2*_ *S. haematobium ITS2*: Sh × ShSb) per area. Total number (percentage in parentheses) of *S. bovis* (Sb) and *S. haematobium* (Sh) *cox1* haplotype and *ITS2* alleles per site.

Sites	Sb × SbSb	Sh × ShSh	Sb × ShSb	Sb × ShSh	Sh × SbSb	Sh × ShSb	Total hybrids	All total	*Cox1* haplotypes		*ITS2* alleles	
	*n* (%)	*n* (%)	*n* (%)	*n* (%)	*n* (%)	*n* (%)	*n* (%)		Sb	Sh	Sb	Sh
Adzopé	3 (0.6)	101 (20.4)	47 (9.5)	320 (64.7)	4 (0.8)	20 (4.0)	391 (79.0)	495	370 (74.7)	125 (25.3)	81 (8.2)	909 (91.8)
Agboville	9 (1.8)	170 (33.9)	59 (11.8)	90 (18.0)	34 (6.8)	139 (27.7)	322 (64.3)	501	158 (31.5)	343 (68.5)	284 (28.4)	718 (71.6)
Sikensi	3 (0.49)	311 (51.0)	49 (8.0)	184 (30.2)	2 (0.33)	61 (10.0)	296 (48.5)	610	236 (46.3)	374 (53.7)	120 (9.8)	1100 (90.2)
Duekoué	0	209 (37.5)	19 (3.4)	314 (56.3)	3 (0.54)	13 (2.33)	349 (62.5)	558	333 (59.7)	225 (40.3)	38 (3.4)	1078 (96.6)
Total	15 (0.7)	791 (36.6)	174 (8.0)	908 (42.0)	43 (2.0)	233 (10.8)	1358 (62.7)	2 164	1097 (50.7)	1067 (49.3)	523 (12.1)	3805 (87.9)

*χ*^2^ test of difference of relative frequencies between sampling sites: *χ*^2^ = 555.9; *df* = 15; *p* < 0.0001. Binomial test of *S. bovis* vs. *S. haematobium* allele’s equipartition: *Cox1*, *p* = 0.27; *ITS2*, *p* < 0.00001.

### Genetic diversity

Of the 2164 miracidia from which *cox1*/*ITS2* profiles were obtained, 1966 (90.8%) also produced reliable allele data for at least 10 microsatellite loci for subsequent analyses. We found a significant genotypic disequilibrium (adjusted *p*-value at 5% level < 0.001) for 120 pairwise locus combinations across the whole dataset. Genetic diversity indices (*He*, *A*, *Ar* and *F*_IS_) and the probability of deviation from Hardy–Weinberg equilibrium (*P*_HWE_) for each microsatellite locus by sampling site are shown in Table 2. Most loci showed a deviation from Hardy–Weinberg equilibrium. All loci were highly polymorphic and the number of alleles and the allelic richness ranged from 7 to 17 and 7.0 to 15.8, respectively per locus in the whole dataset. Inter-population comparisons showed that heterozygosity (*He*) was relatively stable (Friedman test, χ^2^ = 1.33, degree of freedom (*df* = 3, *p* = 0.72) and that allelic richness was more variable ranging between 8.8 and 10.5 for Sikensi and Adzopé, respectively. A significant difference was observed for this last parameter (Friedman test, χ^2^ = 15.33, *df* = 3, *p* = 0.002), allelic richness from Duekoué being statistically higher compared to the remaining three sites (Nemeyi test, *p* < 0.05). No differences in genetic diversity indices were observed according to the different *cox1/ITS2* genetic profiles.

**Table 2:** Genetic diversity indices. Mean expected heterozygosity (*He*), total number of alleles detected (*A*), allelic richness (*Ar*) rarefied to 362 diploid individuals per population, mean inbreeding coefficient (*F*_IS_), and the probability of deviation from Hardy–Weinberg equilibrium (*P*_HWE_) for each microsatellite locus per site. *n*, number of miracidia genotyped per site.

Locus	Adzopé (*n* = 440)					Agboville (*n* = 465)					Duekoué (*n* = 527)					Sikensi (*n* = 534)					Total (*n* = 1966)		
	*He*	*A*	*Ar*	*F*_IS_	*P*_HWE_	*He*	*A*	*Ar*	*F*_IS_	*P*_HWE_	*He*	*A*	*Ar*	*F*_IS_	*P*_HWE_	*He*	*A*	*Ar*	*F*_IS_	*P*_HWE_	*He*	*A*	*Ar*
Sh9	0.72	9	8.86	0.37	<0.001	0.58	11	10.49	0.41	<0.001	0.78	12	11.67	0.50	<0.001	0.54	10	8.68	0.33	<0.001	0.66	14	10.98
Sh3	0.83	14	13.96	0.20	<0.001	0.78	15	14.60	0.11	<0.001	0.88	16	15.53	0.08	<0.001	0.83	14	13.47	0.01	0.836	0.83	16	15.00
C102	0.44	4	3.84	0.10	0.220	0.46	6	5.76	−0.06	<0.001	0.43	7	6.70	0.08	0.819	0.50	5	4.66	−0.06	<0.001	0.46	7	6.05
Sh1	0.77	9	8.88	0.01	<0.001	0.68	9	8.55	−0.01	<0.001	0.75	12	11.97	0.03	<0.001	0.74	8	7.99	−0.03	<0.001	0.74	13	11.85
Sh14	0.81	14	14.00	0.04	<0.001	0.83	14	13.84	0.03	<0.001	0.83	15	14.63	0.00	<0.05	0.82	16	15.56	−0.01	<0.001	0.82	17	15.78
Sh6	0.53	7	6.98	0.24	<0.001	0.58	7	6.99	0.07	<0.001	0.48	7	7.00	−0.01	<0.001	0.60	7	6.99	−0.11	<0.001	0.55	7	7.00
C111	0.55	4	4.00	0.18	<0.001	0.44	5	5.00	0.11	<0.001	0.65	8	7.59	0.03	0.276	0.56	8	7.44	0.01	0.688	0.55	9	7.12
Sh7	0.68	7	6.83	0.26	<0.001	0.74	5	5.00	0.32	<0.001	0.64	7	6.99	0.31	<0.001	0.64	5	4.74	0.32	<0.001	0.68	7	6.69
Sh13	0.66	15	14.67	0.01	<0.001	0.51	14	13.37	0.01	<0.001	0.69	15	14.52	−0.02	<0.001	0.71	14	13.64	−0.03	0.408	0.64	17	15.68
Sh4	0.74	9	9.00	0.05	<0.01	0.73	9	8.92	0.08	<0.001	0.80	11	10.82	0.04	<0.001	0.85	9	9.00	0.07	<0.001	0.78	11	10.22
Sh11	0.51	7	6.62	−0.01	<0.001	0.68	6	5.81	0.12	<0.001	0.46	8	7.54	0.13	<0.001	0.70	8	7.66	0.09	<0.001	0.59	10	7.80
Sh15	0.49	7	7.00	0.16	<0.001	0.54	6	6.00	0.17	<0.001	0.50	6	5.97	0.13	<0.001	0.46	6	6.00	−0.02	<0.001	0.50	7	7.00
Sh2	0.78	13	13.00	0.35	<0.001	0.80	12	12.00	0.32	<0.001	0.89	14	13.96	0.45	<0.001	0.86	12	12.00	0.35	<0.001	0.83	15	14.64
Sh5	0.73	10	9.90	0.44	<0.001	0.47	13	12.34	0.26	<0.001	0.80	15	14.70	0.10	<0.001	0.64	9	8.73	0.16	<0.001	0.66	16	13.95
Sh10	0.48	8	7.85	0.51	<0.001	0.53	6	5.79	0.35	<0.001	0.48	8	7.88	0.31	<0.001	0.33	7	6.64	0.10	<0.001	0.46	8	7.56
Sh12	0.10	4	3.83	−0.04	0.972	0.29	6	5.95	0.03	<0.001	0.07	7	6.06	−0.03	1.0	0.23	8	7.58	−0.06	<0.001	0.17	10	7.68
Mean	0.61	8.81	8.70	0.18	−	0.60	9.00	8.78	0.15	−	0.63	10.50	10.22	0.13	−	0.63	9.13	8.80	0.07	−	0.62	11.50	10.31
SE	0.19	3.60	3.61	0.17	−	0.15	3.61	3.47	0.14	−	0.22	3.61	3.59	0.17	−	0.18	3.28	3.22	0.15	−	0.18	3.88	3.65

*SE*: Standard error*.*

### Population genotypic differentiation

The pairwise *F*_ST_ values between sites ranged from 4.1% to 6.9% with Duekoué being consistently most differentiated from the other sites (Table 3). The *F*_ST_ values between the samples that presented these *cox1*/*ITS2* profiles ranged from 0.3% to 2.7% and thus showed that the *S. bovis cox1-ITS2* profiles (*Sb* × *SbSb*) were not different from the other *cox1*/*ITS2* profiles (Table 4). The miracidia with the *S. bovis cox1* × *S. bovis ITS2* (*Sb* × *SbSb*) genetic profile came from three sites (7 from Adzopé, 4 from Agboville, and 4 from Sikensi) (Supplementary Tab. S2).

**Table 3:** Pairwise estimates of FST (below the diagonal) and significance (above diagonal) between parasite populations of the four sites based on 16 microsatellite loci.

Sites	Adzopé	Agboville	Duekoué	Sikensi
Adzopé	–	**	**	**
Agboville	0.049	–	**	**
Duekoué	0.069	0.065	–	**
Sikensi	0.047	0.041	0.056	–

**Significant values at *p* < 0.01.

**Table 4:** Pairwise estimates of *F*_ST_ (below the diagonal) and significance (above diagonal) between *cox1-ITS2* profiles of the four sampling sites based on 16 microsatellite loci.

Genotype	Sb_SbSb	Sb_ShSb	Sb_ShSh	Sh_SbSb	Sh_ShSb	Sh_ShSh
Sb_SbSb	–	NS	*	*	NS	NS
Sb_ShSb	0.003	–	*	*	*	*
Sb_ShSh	0.011	0.0071	–	*	*	*
Sh_SbSb	0.015	0.023	0.027	–	*	*
Sh_ShSb	0.006	0.005	0.014	0.009	–	*
Sh_ShSh	0.004	0.003	0.006	0.021	0.008	–

NS: not statistically significant; *Statistically significant at the *p* < 0.05 level.

**Figure 2:**
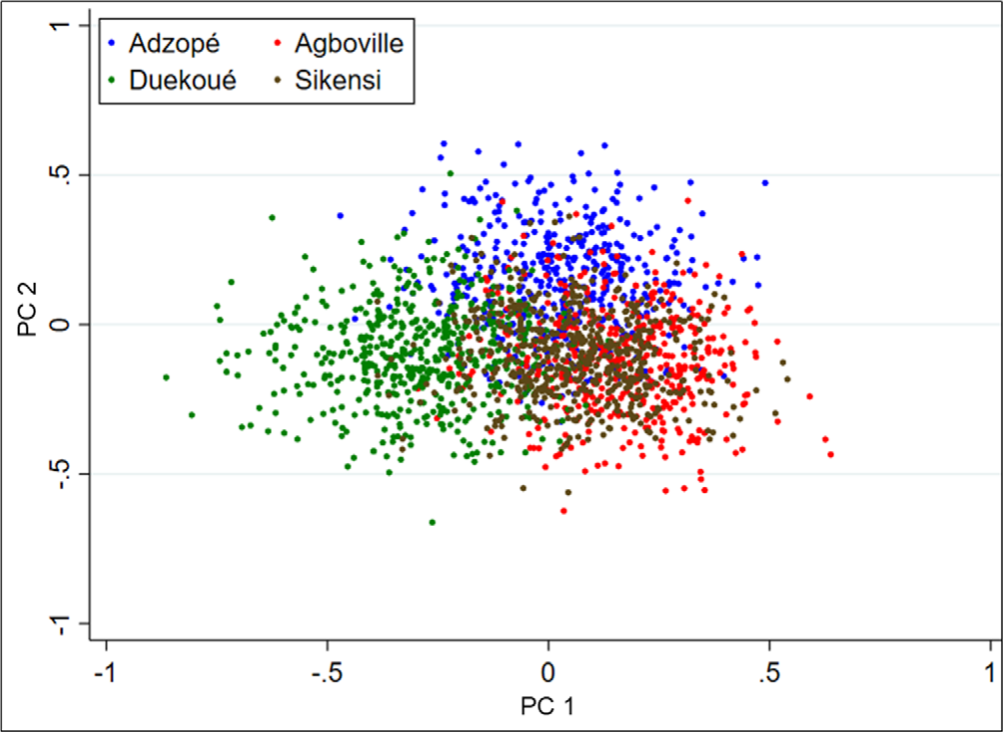
Principal component analysis (PCA) of microsatellite data by sites. Each miracidium is represented by one dot. The first two principal components (PCs) explain 41.1% and 33.7% of total inertia of the data set, respectively.

**Figure 3:**
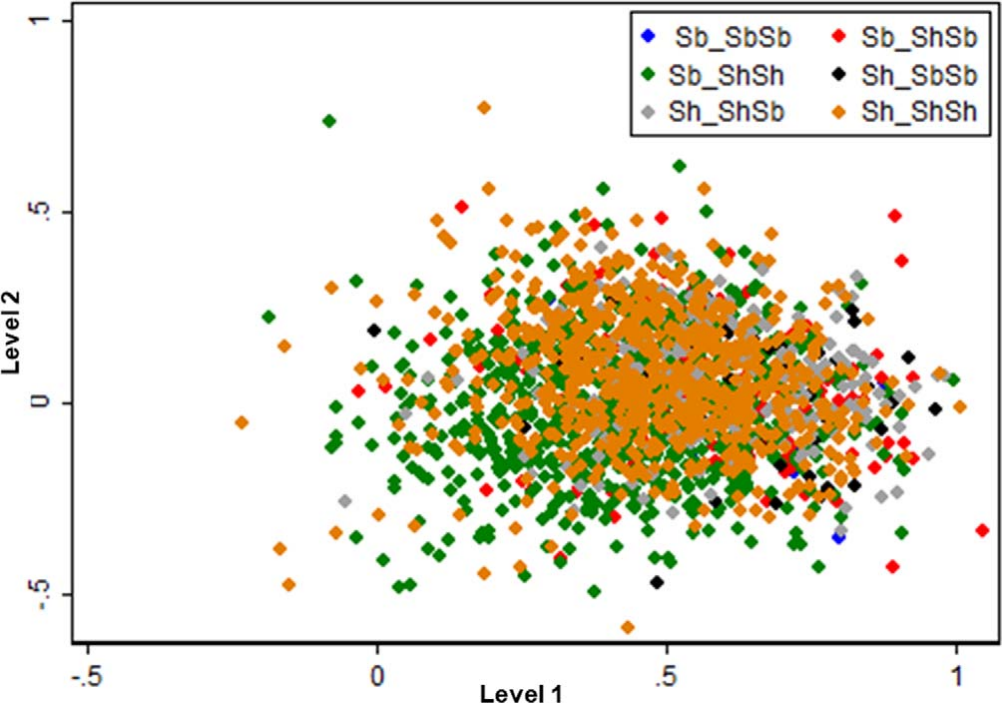
Principal component analysis (PCA) of the microsatellite data with each miracidium assigned its *cox1-ITS2* genotype. Each miracidium is represented by a single dot. The first two principal components (PCs) explain 61.6% and 21.0% of total inertia of the data set, respectively.

The PCA revealed only weak structuration among the four sites with only Duekoué being partially separated from the other three sites (Fig. 2). Comparing the different genetic profile, the PCA showed no genetic structuring according to the six different genetic profiles (Fig. 3).

**Figure 4:**
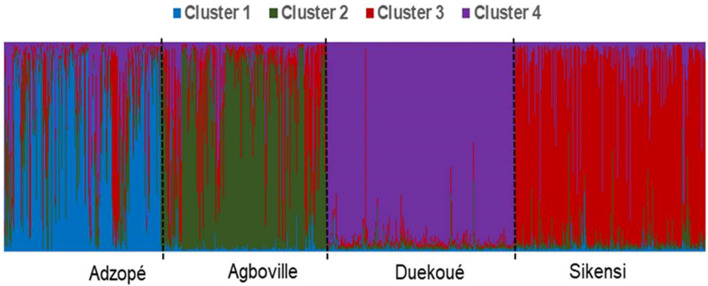
Bar plot depicting the genetic population structure of 1966 *Schistosoma* miracidia from the four sampling sites in Côte d’Ivoire produced by Structure for *K* = 4. Each column represents one miracidium, with colors indicating the proportional contribution of each of the four identified genetic clusters.

Population genetic structure was assessed both at the population level (between sampling sites) and at the infrapopulation level (between children within each sampling site – see below). *K* = 4 displayed maximal delta *K* at the population level suggesting that all miracidia were grouped into four genetic clusters. The Structure analysis showed a relatively strong genetic structure between sampling sites (Fig. 4). At the population level, an average of 51% of the genotypes were assigned to the respective dominant cluster in Adzopé, 63% in Agboville, 69% in Sikensi, and 83% in Duekoué.

### Infrapopulation genotypic differentiation

**Figure 5:**
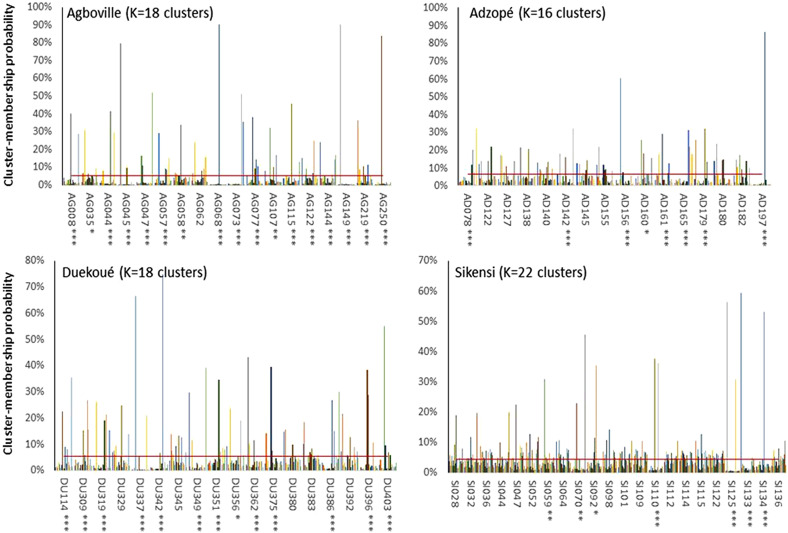
Genetic clustering produced by Structure software for each population with *K* = number of patients. The patient codes are in abscises. *, ** and *** represent statistical difference from random repartition among the clusters at the 5%, 1% and 0.1% levels, respectively. The red line represents the null hypothesis of random repartition (1/*K*).

**Supplementary Fig. S2:**
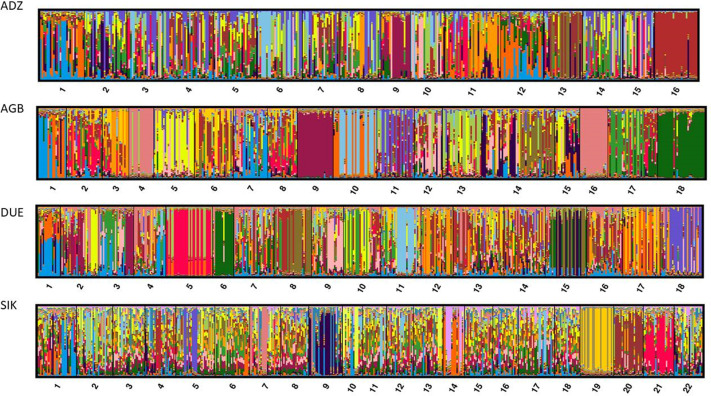
Bar plots depicting the percentage for each miracidium belonging to each cluster by child, using children hosting at least 15 miracidia in Adzopé (ADZ), Agboville (AGB), Duekoué (DUE), and Sikensi (SIK). This was done in Structure and each site K was set equal to the number of children.

The analysis at the infrapopulation level revealed 16–22 clusters at all sites; reflecting the individual children sampled. The Structure analysis for each population with *K* = number of patients showed that no best *K* was found for all sampling sites. However, the clusters were not uniformly distributed among children and some miracidia were assigned to a restricted number of clusters (Fig. 5). This non-random repartition pattern was significant for 60.8% of the infrapopulations analysis (13/18 for Duekoué, 7/22 for Sikensi, 17/18 for Agboville and 8/16 for Adzopé). Supplementary Fig. S2 shows bar plots depicting the probability for each miracidium to belong to each cluster by child, using only those children who hosted at least 15 miracidia.

The analysis of molecular variance (AMOVA) showed that most of the miracidial genetic variance was found within sites (57.8% of the variance). There is more variance among miracidia by site rather than by host (10.4%) (Table 5).

**Table 5:** Analysis of molecular variance (AMOVA) partitioning the total residual variance (after deducting the non-informative variance between microsatellite loci) observed at 16 microsatellite loci in 1966 miracidia between four hierarchical levels.

Hierarchical level	*df*	Sum of squares	Variance component	Percentage of residual variation
Among sites	3	25,659	7.83	31.8
Among children within sites	87	68,119	14.26	57.8
Among miracidia within child	1875	320,706	2.78	10.4
Within miracidia (between microsatellite loci)	1966	326,144	165.89	–
Total	3931	740,628	190.55	–

*df*: degrees of freedom.

## Discussion

Our study showed a high proportion of *S. haematobium* × *S. bovis* hybrids among school-aged children in different parts of Côte d’Ivoire. Similar results were also reported from a large study in Senegal, where most children (88%) excreted hybrid miracidia [64]. Notably, virtually all children (97.8%) were producing miracidia with a hybrid genotype (i.e. a discrepancy between the mitochondrial and the nuclear signature and/or heterozygous nuclear profiles). Moreover, we found no genetic differentiation of miracidia presenting *S. haematobium, S. bovis* or hybrid genetic profiles, which indicates that all genotyped parasites most likely belong to a single genetic entity. This finding is in line with a recent genomic study indicating that most, if not all *S. haematobium* lineages across Africa, are likely to be introgressed to some extent with *S. bovis* [48].

We assigned all miracidia to six distinct *cox1-ITS2* profiles, of which four were unambiguous hybrid profiles. Hybrids with a mitochondrial *cox1* haplotype from *S. bovis* and homozygous *ITS2* from *S. haematobium* (*Sb* × *ShSh*) were the most commonly identified type at 41.9%. Such hybrids have sometimes been documented in Senegal and ascribed to bidirectional introgressive hybridization [33, 63]. Beside this general pattern, significant variation was observed between sampling sites with the highest frequency of hybrids (reaching 79%) identified in Adzopé. Interestingly, the genetic contribution of the *S. bovis* genetic profile to the genetic make-up of schistosome populations is much more evident at the mitochondrial level than at the nuclear level. The frequency of miracidia with a heterozygous *ITS2*, i.e. carrying both *S. bovis* and *S. haematobium ITS* alleles, was relatively low, which could be explained by the concerted evolution of *ITS2* gene region [25].

The *S. bovis cox1* × *S. bovis ITS2* genetic profile (SbSb) has also been reported in miracidia recovered from humans in Corsica [10]. We only detect 0.7% of this profile in our study. Importantly, this genetic profile using only two gene genotyping methods does not mean that these parasites are “pure” *S. bovis* parasites at the whole genome level. The genetic signature can only represent a certain introgression level between both species, and can explain some discrepancy in parasite phenotype such us the route of excretion expected to be in the feces for the *S. bovis* parasite.

Data on the genetic structure of *S. haematobium* populations are sparse [29, 30]. Previous findings based on a phylogeographic approach, using *cox1* data, at the African continental scale suggested that *S. haematobium* is less structured than *S. mansoni* [63] and *S. bovis* [48]. In contrast, the significant differentiation between sampling sites and even within individual hosts reported here, suggests a genetic structure of miracidia at small geographical scales. The large geographical distance separating Duekoué in the western part of Côte d’Ivoire to the other three sites is likely to limit mixing between these populations and lead to genetic structuring between the schistosome populations. More surprising is the genetic structure observed at the individual level. In the majority of cases, each child hosted an infrapopulation different from the other children. Our finding does not corroborate the “genetic mixing bowl hypothesis” [16]. The authors reported that the long-living definitive hosts cumulate genotypes coming from several short-living infected intermediate host snails, which can lead to homogeneity of infrapopulations. The pattern found in our study could be explained by recent treatment of children by praziquantel one year before sample collection. In this scenario, children have only recently started to harbour parasites again and there was insufficient time for “mixing” after praziquantel treatment. This would homogenize parasite populations among children rather than promoting structuration. Children would therefore be infected with only one or a few pairs of parasites and they could also be highly exposed and have several infections. This clustering may be amplified by the genotypic specific immune responses. In a mouse model, it was found that a trickle infection (repeated light infections) can protect mice against a *Schistosoma* challenge infection [20]. Subsequently, the same authors have shown that this level of protection is genotype-dependent and correlated with genetic dissimilarity between the immunizing and the challenging infection [20]. The smaller the genetic distance between immunizing and challenging clones, the lower the infectivity rate of the challenging clones. This mechanism may, in turn, increase genetic diversity in each host and differentiate the parasite infrapopulation between hosts. No matter what the reasons are for this pattern, it emphasises the importance of investigating the parasites from as many different patients (or hosts in general), when investigating population genetics between different sampling sites. Increasing the number of hosts sampled, rather than the number of miracidia per host, has already been proposed after a stochastic re-sampling approach [24].

No genetic differentiation was observed according to the genetic profile. This result suggests that there are no genetic mating restrictions between the six different genetic profiles, and hence, all recombinations are possible [11, 27]. Similar findings have recently been reported from Senegal [11]. So, our results together with recent genomic studies [44, 48], call into question *S. haematobium* and whether forms of hybrids detected in humans thus far are truly different genomic entities.

The current study has some limitations, related to the reliability of the RFLP analysis, the absence of samples from animals, or the hybrid genotyping method. First, in our study we used an RFLP method based on a short 505 bp *ITS2* fragment. Even though the results we obtained are robust, the RFLP method is known to be sensitive to different factors such as enzyme used, and the time or temperature of digestion. A new method for single-nucleotide polymorphism detection adapted to large scale screening needs to be developed. Second, miracidia from livestock or rodents were not collected. This is an important point to infer the possible zoonotic capacity of these hybrid pathogens. The situations are contrasted depending on the countries without current evidence of zoonotic transmission in Senegal [12] compared to rodent and cow reservoir hosts in Benin [54]. Finding a high frequency of hybrids in Côte d’Ivoire does not confirm zoonotic transmission and we advocate looking for the presence of hybrid schistosomes in rodents and livestock. Third, mito-nuclear genotyping methods are well adapted for large population screening; however, as previously mentioned, the whole genome cannot be resumed to these two gene assignations, and genomic studies using more genetic markers are desirable.

## Conclusions

Our study investigated the genetic diversity and population structuring of schistosomes at the population level. Each child harboured a genetic cluster of schistosomes at the infrapopulation level, which could lead to putative resistant parasite selection due to drug pressure by mass drug administration (MDA). The high frequency of hybrids between *S. haematobium* × *S. bovis* observed can lead to difficulties in accurately diagnosing schistosome hybrids by conventional techniques using light microscopy. This could also have adverse consequences on schistosomiasis control towards elimination and negatively impact disease transmission. More studies are need on population genetics of schistosomes at the human and animal interface to evaluate the parasite’s gene flow.

## Supplementary materials

The Supplementary material of this article is available at https://www.parasite-journal.org/10.1051/parasite/2022023/olm. *Supplementary Tab. S1*: Microsatellite dataset. *Supplementary Tab. S2*: Total number of miracidia analyzed (total) and number (n) and percentage (%) of miracidia assigned to the six possible *cox1*/*ITS2* profiles defined by *cox1*-PCR and *ITS2*-RFLP analysis per child and site.
