# Supplementary material for: Population genetic structure of Schistosoma haematobium and Schistosoma haematobium × Schistosoma bovis hybrids among school-aged children in Côte d’Ivoire
Source: Parasite. 2022 May 3;29:23. doi: 10.1051/parasite/2022023 (PMC9074780; doi:10.1051/parasite/2022023)
Supplement: Supplementary file 1 — Supplementary Tab. S1: Microsatellite dataset. Supplementary Tab. S2: Total number of miracidia analyzed (total) and number (n) and percentage (%) of miracidia assigned to the six possible cox1/ITS2 profiles defined by cox1-PCR and ITS2-RFLP analysis per child and site. [file parasite-29-23-s1.zip › parasite210073-1-olm/parasite210073-2-olm.pdf]

Supplementary Tab. S2: Total number of miracidia analyzed (total) and number (n) and percentage (%) of miracidia assigned to the six possible *cox1*/*ITS2* profiles defined by *cox1*-PCR and *ITS2*-RFLP analysis per child and site.

Areas	Child Id	Sb x SbSb	Sb x ShSb	Sb x ShSh	Sh x SbSb	Sh x ShSb	Sh x ShSh	Total
		n (%)	n (%)	n (%)	n (%)	n (%	n (%)	
Adzopé	AD078	0	3 (10.3)	25 (68.2)	0	0	1 (3.4)	29
	AD088	0	0	1 (12.5)	3 (37.5)	4(50.0)	0	8
	AD091	0	0	1 (14.3)	0	4 (57.1)	2 (28.6)	7
	AD094	0	0	1 (12.5)	0	3 (37.5)	4 (50.0)	8
	AD122	0	8 (28.6)	18 (64.3)	0	0	2 (7.1)	28
	AD127	0	1 (4.2)	15 (62.5)	0	0	8 (33.3)	24
	AD128	0	1 (6.2)	9 (56.2)	0	0	6 (37.5)	16
	AD138	0	1 (2.9)	27 (79.4)	0	0	6 (17.6)	34
	AD140	0	0	23 (82.1)	0	0	5 (17.9)	28
	AD142	0	0	22 (73.3)	0	0	8 (26.7)	30
	AD145	1 (3.4)	3 (10.3)	20 (69.0)	0	0	5 (17.2)	29
	AD155	0	5 (17.9)	17 (60.7)	0	0	6 (21.4)	28
	AD156	2 (10.0)	4 (20.0)	6 (30.0)	0	1 (5.0)	7 (35.0)	20
	AD158	0	0	1 (7.1)	0	6 (42.9)	7 (50.0)	14
	AD160	0	1 (4.5)	17 (77.3)	0	0	4 (18.2)	22
	AD161	0	10 (29.4)	9 (26.5)	0	1 (2.9)	14 (41.2)	34
	AD165	0	4 (14.3)	21 (75.0)	0	1 (3.6)	2 (7.1)	28
	AD179	0	1 (3.6)	23 (82.1)	0	0	4 (14.3)	28
	AD180	0	0	23 (88.5)	0	0	3 (11.5)	26
	AD182	0	2 (8.0)	16 (64.0)	1 (4.0)	0	6 (24.0)	25
	AD197	0	3 (10.3)	25 (86.2)	0	0	1 (3.4)	29

	**Total**	**3 (0.6)**	**47 (9.5)**	**320 (64.6)**	**4 (0.8)**	**20(4.0)**	**101 (20.4)**	**495**
Agboville	AG008	1 (4.5)	2 (9.1)	11 (50.0)	0	3 (13.6)	5 (22.7)	22
	AG035	0	6 (24.0)	5 (20.0)	0	7 (28.0)	7 (28.0)	25
	AG044	4 (23.5)	3 (17.6)	4 (23.5)	0	2 (11.7)	4 (23.5)	17
	AG045	0	0	0	1 (5.9)	7 (41.2)	9 (52.9)	17
	AG047	0	0	0	4 (15.4)	15 (57.7)	7 (26.9)	26
	AG057	0	0	2 (7.1)	0	15 (53.6)	11 (39.3)	28
	AG058	0	5 (20.0)	13 (52.0)	0	6 (24.0)	1 (40.0)	25
	AG062	0	2 (10.5)	4 (21.0)	0	7 (36.8)	6 (31.6)	19
	AG066	0	0	0	0	11 (73.3)	4 (26.7)	15
	AG067	0	0	1 (50.0)	0	0	1 (50.0)	2
	AG068	0	0	0	14 (56.0)	3 (12.0)	8 (32.0)	25
	AG073	0	1 (3.6)	0	5 (17.9)	9 (32.1)	13 (46.4)	28
	AG077	2 (8.3)	10 (41.7)	9 (37.5)	0	1 (4.2)	2 (8.3)	24
	AG086	0	0	0	0	3 (25)	9 (75.0)	12
	AG107	0	0	0	0	6 (33.3)	12 (66.7)	18
	AG108	0	0	4 (24.7)	3 (20.0)	2 (13.3)	6 (40.0)	15
	AG115	0	10 (37.0)	12 (44.4)	0	2 (7.4)	3 (11.1)	27
	AG122*	2 (4.0)	13 (26.0)	8 (16.0)	2 (4.0)	7 (14.0)	18 (36.0)	50
	AG144	0	1 (5.3)	12 (63.2)	0	2 (10.5)	4 (21.0)	19
	AG149	0	1 (4.8)	0	0	11 (52.4)	9 (42.9)	21
	AG219	0	4 (12.1)	4 (12.1)	5 (15.1)	12 (36.4)	8 (24.2)	33
	AG250	0	1 (3.0)	1 (3.0)	0	8 (24.2)	23 (69.7)	33
	**Total**	**9 (1.8)**	**59 (11.8)**	**90 (18.0)**	**34 (6.8)**	**139 (27.7)**	**170 (33.9)**	**501**
Sikensi	SI028	1 (2.9)	3 (8.8)	9 (26.5)	0	4 (11.8)	17 (50.0)	34
	SI032	0	0	5 (17.9)	0	7 (25.0)	16 (57.1)	28
	SI036	0	4 (14.3)	4 (14.3)	0	9 (32.1)	11 (39.3)	28

	SI044	0	0	15 (57.7)	0	0	11 (42.31)	26
	SI047	0	1 (3.1)	11 (34.4)	0	0	20 (62.5)	32
	SI052	0	9 (31.0)	5 (17.2)	0	4 (13.8)	11 (37.9)	29
	SI059	0	0	3 (13.0)	0	0	20 (87.0)	23
	SI064	0	0	9 (37.5)	0	8 (33.3)	7 (27.2)	24
	SI070	0	0	3 (12.0)	1 (4.0)	0	21 (84.0)	25
	SI075	0	1 (4.8)	4 (19.0)	0	3 (14.3)	13(61.9)	21
	SI078	0	3 (42.9)	3 (42.9)	0	0	1 (14.3)	7
	SI091	0	0	2 (15.4)	0	1 (7.7)	10 (76.9)	13
	SI092	0	0	4 (22.2)	0	4 (22.2)	10 (55.6)	18
	SI098	0	2 (9.5)	4 (19.0)	1 (4.8)	2 (9.5)	12 (57.1)	21
	SI101	0	1 (4.5)	12 (54.5)	0	2 (9.1)	7 (31.8)	22
	SI104	0	2 (13.3)	6 (40.0)	0	1 (6.7)	6 (40.0)	15
	SI109	0	2 (8.3)	11 (45.8)	0	1 (4.2)	10 (41.7)	24
	SI110	0	0	0	0	2 (10.5)	17 (89.5)	19
	SI112	0	1 (4.2)	8 (33.3)	0	3 (12.5)	12 (50.0)	24
	SI114	0	2 (8.0)	10 (40.0)	0	0	13 (52.0)	25
	SI115	0	2 (6.9)	11 (37.9)	0	1 (3.4)	15 (51.7)	29
	SI122	0	3 (14.3)	6 (28.6)	0	5(23.8)	7 (33.3)	21
	SI125	0	0	0	0	1 (3.6)	27 (96.4)	28
	SI133	0	7 (30.4)	14 (60.9)	0	0	2 (8.7)	23
	SI134	0	4 (15.4)	20 (76.2)	0	0	2 (7.7)	26
	SI136	2 (8.0)	2 (8.0)	5 (20.0)	0	3(12.0)	13 (52.0)	25
	**Total**	**3 (0.5)**	**49 (8.0)**	**184 (30.2)**	**2 (0.3)**	**61 (10.0)**	**311 (50.9)**	**610**
Duekoué	DU053	0	0	13 (100.0)	0	0	0	13
	DU114	0	5 (29.4)	11(64.7)	0	0	1 (5.9)	17
	DU197	0	0	0	1 (25.0)	3 (75.0)	0	4

DU309	0	0	11 (34.4)	1 (3.1)	2 (6.2)	18 (56.2)	32
DU319	0	0	6 (21.4)	0	2 (7.1)	20 (71.4)	28
DU326	0	0	10 (76.9)	0	0	3 (23.1)	13
DU329	0	3 (10.3)	8 (27.6)	0	2 (6.9)	16 (55.2)	29
DU330**	0	0	0	0	0	2 (100.0)	2
DU337**	0	0	0	0	0	35 (100.0)	35
DU342	0	0	15 (78.5)	0	0	4 (21.0)	19
DU345	0	0	19 (59.4)	0	0	13 (40.6)	32
DU349	0	0	17 (56.7)	0	0	13 (43.3)	30
DU351	0	0	12 (48.0)	0	0	13 (52.0)	25
DU356	0	2 (7.1)	6 (21.4)	0	4 (14.3)	16 (57.1)	28
DU362	0	0	20 (62.5)	0	0	12 (37.5)	32
DU375	0	1 (4.0)	21 (84.0)	0	0	3 (12.0)	25
DU380	0	2 (5.3)	23 (60.5)	0	0	13 (34.2)	38
DU383	0	1 (3.2)	18 (58.1)	0	0	12 (38.7)	31
DU386	0	0	28 (87.5)	0	0	4 812.5)	32
DU392	0	2 (6.4)	0	2 (6.4)	27 (87.1)	0	31
DU396	0	6 (13.4)	0	3 (9.7)	22 (77.0)	0	31
DU403	0	0	27 (87.1)	1 (3.2)	0	3 (9.7)	31
**Total**	**0**	**19 (3.4)**	**314 (56.3)**	**3 (0.5)**	**13 (2.3)**	**209 (37.5)**	**558**
**All total**	**15 (0.7)**	**174 (8.0)**	**908 (42.0)**	**43 (2.0)**	**233 (10.8)**	**791**	**2 164**

*: Infected by parasites of all the six possible *Cox1-ITS2* profiles. **: Infected by only "pure" parasite genotypes.
